# A perspective on HPK1 as a novel immuno-oncology drug target

**DOI:** 10.7554/eLife.55122

**Published:** 2020-09-08

**Authors:** Sansana Sawasdikosol, Steven Burakoff

**Affiliations:** Tisch Cancer Institute, Department of Oncological Sciences, Icahn School of Medicine at Mount Sinai, Hess Center for Science and MedicineNew YorkUnited States; PfizerUnited States; PfizerUnited States

**Keywords:** tumor immunity, immunotherapy, negative regulator of immune response

## Abstract

In this perspective review, the role Hematopoietic Progenitor Kinase 1 (HPK1) in tumor immunity will be reviewed, with special emphasis on how T cells are negatively-regulated at different junctures of cancer-immunity cycle by this regulatory kinase. The review will highlight the strengths and weaknesses of HPK1 as a candidate target for novel immuno-oncology (IO) drug development that is centered on the use of small molecule kinase inhibitor to modulate the immune response against cancer. Such a therapeutic approach, if proven successful, could supplement the cancer cell-centric standard of care therapies in order to fully meet the therapeutic needs of cancer patients.

## Introduction

The antibody-based immune checkpoint inhibitor drugs are likely to remain one of the most effective platforms for IO drug development for the foreseeable future, given that they are capable of providing a significant percentage of cancer patients with durable anti-tumor responses; thus, improving survival rates compared to those treated with traditional standard of care therapies (Ribas and Wolchok, 2018). However, the response rate to IO therapy varies widely among tumor types - ranging from a low of 15% objective response rate for Head and Neck cancer patients to a high of 87% objective response rate for patients with Hodgkin’s Disease (Ribas and Wolchok, 2018). The lack of broad therapeutic efficacy highlights the need to identify biomarkers capable of predicting either which type of cancer or which biomarker sub-stratums would respond best to the therapy (Chen and Mellman, 2017; Havel et al., 2019). In addition, there is a need to develop other immune checkpoints such as TIM-3 (Das et al., 2017; Joller and Kuchroo, 2017), LAG-3 (Anderson et al., 2016; Maruhashi et al., 2018), VISTA (ElTanbouly et al., 2020), CEACAM1 (Khairnar et al., 2018; Dankner et al., 2017) and TIGIT (Manieri et al., 2017; Dougall et al., 2017) to broaden the scope of therapeutic coverage by the current repertoire of checkpoint inhibitor drugs (Le Mercier et al., 2015; Marhelava et al., 2019). It is reasonable to argue that, in addition to identifying novel antagonistic antibodies that recognize other checkpoint inhibitor axes, alternative approaches to modulate T cell immune responses should also be considered.

Several small molecule kinase inhibitors that block specific pathways involved in the regulation of tumor immunity have been proposed (Adams et al., 2015; Weinmann, 2016; Sasikumar and Ramachandra, 2018). The advantage of this approach is the fact that the development of these drugs relies on the use of mature technologies that have a decades-long proven track record of success. Large commercial and proprietary libraries of small molecule compounds exist and the process of high throughput screening to identify specific kinase inhibitors is well-developed. Cell-permeable lead compounds that would emerge from these screens could be subjected to further refinement through medicinal chemistry to improve specificity, potency, bioavailability and other drug characteristics through iterative structure-activity relation (SAR) analysis. This process could be further facilitated by the pre-existing collection of three-dimensional structures of the many kinase domains, making rational structure-based drug design possible. The challenge of using this approach to develop new IO drugs lies in determining which of the more than 500 kinases in the human genome would be appropriate targets (Manning, 2002).

## Candidates for small molecule kinase inhibitor-based IO drugs

Identifying an ideal kinase that is involved in negative regulation of T cell responses to cancer pose a serious vetting challenge for the small molecule-based IO drug discovery program. One approach to selecting a drug candidate with the best potential to succeed is to select a kinase that plays multiple negative regulatory roles at different stages of the ‘cancer-immunity cycle’. The cancer-immunity cycle is a term used to describe an intricate, multi-step process involving interactions between T cells, other immune cells, cancer cells and epithelial cells throughout the initiation and execution phases of anti-cancer immune responses (Chen and Mellman, 2013). In addition to the roles such a kinase plays in the cancer-immunity cycle, there should be genetic evidence that the kinase activity of the drug candidate is important in the inhibition of an effective anti-cancer immune response. As an additional criterion, a preference should be given in selecting a kinase with an immune cell-restricted expression profile. This criterion might minimize the occurrence of adverse effects caused by an on-target inhibition in non-immune cell types where the kinase might serve a critical non-immune cell-related functions. Taken together, these characteristics could be used as a framework to guide the selection of target candidates that are most likely to succeed as small molecule kinase inhibitor-based IO drugs.

## Hematopoietic progenitor kinase 1 (HPK1) is catalytically activated by ligand engagement of a number of receptors found on T cells and other immune cells

T cell antigen receptor (TCR)-generated activation signals involve enzymes, typically kinases, to propagate and amplify these signals (Smith-Garvin et al., 2009; Gaud et al., 2018). *HPK1*, also known as *MAP4K1*, is a hematopoietic cell-restricted member of the *Ste20* family of serine/threonine kinases that functions as a negative regulator of activation signals generated by the TCR (Kiefer et al., 1996; Hu et al., 1996; Liou et al., 2000). The interactions between the protein interacting regions of HPK1 with adaptor proteins have been shown to be crucial to its ability to catalytically respond to the TCR-induced and other non-kinase receptor-induced activation signals (Chuang et al., 2016; Zhang et al., 2017). In order to gain a better understanding of how interfering with HPK1 kinase activity might impact the function of T cells and other immune cells, it is important to first review how HPK1 is activated by various receptors that are expressed on immune cells.

### TCR activation of HPK1

Cell biological studies reveal that HPK1 is catalytically responsive to activation signals generated by a variety of cell surface receptors that are present on many sub-types of hematopoietic cells, including: the TCR (Liou et al., 2000), BCR (Liou et al., 2000; Sauer et al., 2001; Tsuji et al., 2001; Han et al., 2003), Fas (Chen et al., 1999), EPOR (Nagata et al., 1999), TGFβR (Wang et al., 1997; Zhou et al., 1999), PGE_2_’s EP2R and EP4R (Sawasdikosol et al., 2003; Sawasdikosol et al., 2007) and LPS receptors, presumably via TLR2 or TLR4 (Alzabin et al., 2009). Among the receptors that regulate HPK1 kinase activity, signal transduction mechanisms used by TCR to activate HPK1 is best understood. Upon TCR engagement, HPK1 is recruited to the cholesterol- and sphingomyelin-rich lipid raft microdomain via the interactions with LAT, and Gads (Liou et al., 2000; Ling et al., 2001). Once complexed with LAT, HPK1 is phosphorylated on tyrosine 379 by ZAP-70, creating the high affinity binding site for the SH2 domain of SLP-76. This interaction between HPK1 and SLP-76, along with the autophosphorylation of HPK1 at residue threonine 165 and at a PKD-mediated phosphorylation site at serine 171, are required for HPK1 to achieve full catalytic activity (Sauer et al., 2001; Arnold et al., 2005). Once HPK1 becomes catalytically active, HPK1 carries out its role as a negative regulator of TCR-induced signal transduction by phosphorylating SLP-76 at serine residue 376 and Gads at threonine residue 262 (Di Bartolo et al., 2007; Lasserre et al., 2011). These phosphorylated residues form consensus phosphorylation-dependent binding sites for 14-3-3 for each of these LAT signalosome members. The association between phosphorylated SLP-76 and 14-3-3 destabilizes the interaction between SLP-76 and the LAT signalosome (Di Bartolo et al., 2007; Lasserre et al., 2011). The dissociated SLP-76 then undergoes phosphorylation-dependent ubiquitination at its lysine 30 residue, leading to its subsequent proteasome-mediated proteolysis (Wang et al., 2012a). In this model, HPK1 serves as one of the negative feedback loops that limits the strength and duration of TCR signals by disrupting the SLP-76/LAT signalosomes.

### BCR activation of HPK1

HPK1 also functions as a negative regulator of BCR-induced B cell activation, as evidenced by the elevated response profile of ERK, p38, JNK MAPK, PLCγ1, calcium signaling and IKK signaling upon BCR engagement in *HPK1^-/-^* B cells (Wang et al., 2012b). The mechanisms used by BCR to activate HPK1 is nearly identical to the one used by TCR. BCR engages Syk and Lyn tyrosine kinases to activate HPK1, presumably via phosphorylation of tyrosine residue 379, which creates the activation-dependent association between tyrosine phosphorylated HPK1 and the SH2 domain of BLNK (Sauer et al., 2001; Tsuji et al., 2001). The phosphorylation of BLNK’s threonine residue 152 creates the docking site for 14-3-3 and induces ubiquitination at Lys37, Lys-38, and Lys-40 of BLNK, leading to the subsequent degradation of BLNK and the dampening of BLNK-dependent BCR activation signals (Wang et al., 2012b). Thus, through a similar mechanism, BCR also engages HPK1 as a negative feedback mechanism to control B cell activation.

### EP2 and EP4 activation of HPK1

The catalytic activity of HPK1 is responsive to the signals generated by the binding of prostaglandin E2 (PGE_2_) to the rhodopsin-type G protein–coupled receptors, E-prostanoid receptor 2 (EP2) and 4 (EP4) that are present on T cells (Sawasdikosol et al., 2003). PGE_2_ is the primary eicosanoid product generated when cyclooxygenase 2 (COX-2) catalyzes the conversion of arachidonic acid into PGE_2_ – a pluripotent pro-inflammatory lipid mediator capable of inhibiting TCR-induced T cell activation (Vercammen and Ceuppens, 1987). In the context of the immune response to cancer, lung, colon and breast cancers are some of the cancer sub-types that overexpress COX-2 (Wang and DuBois, 2008; Nie, 2007; Hosomi et al., 2000). The high levels of PGE_2_ produced by these COX-2-over-producing cancers contribute substantially to the tumor-generated suppression that prevent the immune system from engaging these cancers effectively (Greenhough et al., 2009; Hashemi Goradel et al., 2019).

How HPK1 is activated by PGE_2_ is not well-understood, but there is evidence that the activation mechanism is quite different from those used by TCR to activate HPK1 (Sawasdikosol et al., 2003; Sawasdikosol et al., 2007). As with activation of HPK1 via TCR, serine 171 of HPK1 needs to be phosphorylated for HPK1 to be catalytically active. However, instead of serine 171 being phosphorylated by PKD, PGE_2_-induced activation of HPK1 utilizes PKA to phosphorylate serine 171 (Sawasdikosol et al., 2007). PGE_2_ could activate HPK1 in mutant Jurkat T cells lines that are deficient in either ZAP-70, LAT or SLP-76, suggesting that EP2 and EP4 receptors utilize a distinct signal transduction cascade from that used by the TCR to activate HPK1 (Sawasdikosol et al., 2007). To date, it remains unknown whether HPK1 undergo PGE_2_-inducible association or dissociation with other signaling molecules or which molecule serve as its kinase substrate.

### Caspase-mediated regulation of HPK1 activity and functions

A third mechanism of HPK1 activation occurs in response to CD95 (Fas) engagement (Chen et al., 1999). Fas, a member of tumor necrosis factor receptor superfamily, plays an important role in the regulation of immune cell functions via the induction of an apoptosis cascade (Wilson et al., 2009). Upon Fas engagement, the adaptor molecule FADD is recruited to Fas and forms the death-inducing signaling complex with procaspase-8 and procaspase-10 (Dickens et al., 2012). Activation of this caspase cascade leads to proteolytic cleavage of HPK1 at aspartic acid residue 385, which is located within the DDVD consensus caspase recognition site of HPK1 (Chen et al., 1999). Caspase-processed HPK1 forms a 43 kD kinase domain-containing N-terminal fragment and a 54 kD C-terminal fragment containing three proline-rich regions and the citron homology domain (Chen et al., 1999). The cleaved N-terminal kinase domain acquires a more hyperactive and sustained kinase activity than the non-cleaved full length HPK1 while retaining its ability to activate the JNK MAPK pathway (Arnold et al., 2007). The association between HPK1 kinase activity and the activation of JNK MAPK pathway had been previously established (Kiefer et al., 1996; Hu et al., 1996) and ectopic expression studies using dominant interfering mutants that function upstream of JNK in hematopoietic and epithelial cell lines revealed that the kinase activity of HPK1 activate the JNK pathway via the activation of the MEKK1/MKK/JNK signaling cascade (Hu et al., 1996; Chen and Tan, 2000). While a Fas-induced increase in JNK activity is entirely consistent with the finding that JNK activity may play a role in apoptosis (Xia et al., 1995), whether the HPK1-induced JNK kinase activity plays a role in Fas-mediated apoptosis warrants further investigation.

The caspase-cleaved N-terminal domain of HPK1 becomes catalytically active upon separation from its C-terminal fragment, which suggest that the C-terminal region of HPK1 is involved in the auto- or trans-regulation of HPK1 kinase activity. This speculation is strengthened by the recent X-ray crystallographic studies revealing that the three-dimensional structure of HPK1 kinase domain exists as a head-to-head domain-swapped dimer in both the crystal form, as well as in the liquid form Wu et al., 2019; Johnson et al., 2019. In the non-phosphorylated, inactive state, the activation loop of each HPK1 monomer partially occupies the ATP- and substrate-binding sites of its partner monomer, thus serving as a possible mechanism to quiesce the basal kinase activity of HPK1. This trans-inhibitory posture of the activation loop is disrupted when the threonine residue 165 and serine residue 171 are phosphorylated (Johnson et al., 2019). This finding demonstrates that the dynamic structural positioning of the HPK1 activation loop is controlled by conformational changes induced by phosphorylation of HPK1. Thus, it is conceivable that the caspase-mediated cleavage that removes the C-terminal domain from the HPK1 kinase domain might induce an enormous change in the energy minimization requirement for dimerization of the N-terminal kinase domain, resulting in the disruption of the domain-swapped trans-inhibitory posture of the activation loop and thus facilitate the activation of the kinase activity of HPK1. Further studies are needed to test the validity of such activation models for HPK1 kinase activity.

The proteolytically cleaved 54 kD C-terminal fragment that contains three proline-rich regions and the citron homology domain has no catalytic activity, but plays an important role in the regulation of apoptosis via its ability to inhibit the NFκB pathway (Chen et al., 1999; Arnold et al., 2001). How the C-terminal HPK1 fragment carries out the negative regulation of the NFκB pathway is best understood from studies of HPK1 regulation of the NFκB pathway in response to TCR engagement. In this setting, HPK1 is also processed by caspase into the two fragments similar to its response to Fas engagement, but at slower temporal kinetics than that induced by Fas. HPK1 is proteolytically cleaved several days after the initial TCR engagement, coinciding with the activation-induced cell death (AICD)-sensitive stage of T cell activation. However, since HPK1 is already activated by TCR via a Lat/Gads/SLP-76-dependent pathway that does not involve proteolytic processing of HPK1, the caspase-mediated processing of HPK1 in this AICD context serves as a mechanism to direct HPK1 substrate specificity. Full length HPK1 had been shown to activate the NFκB pathway during the early, non-AICD-sensitive phase of T cell activation (Arnold et al., 2001; Brenner et al., 2005), through phosphorylation of the linker region of CARMA1 at serine 549 and 551 (Arnold et al., 2001; Brenner et al., 2009). The phosphorylated CARMA1 complexes with Bcl10 and MALT1 to form the CARMA1/Bcl10/MALT1 that phosphorylates and activates IKK kinase activity, resulting in IKK degradation and initiation of the NFκB activation cascade (Brenner et al., 2009). Once HPK1 is cleaved by caspase, its HPK1-C fragment continues to bind to IKKα and IKKβ and sequester them in the inactive form, preventing the initiation of the NFκB cascade (Brenner et al., 2005). Thus, the generation of HPK1-C is akin to a mechanism that blocks the TCR-induced NFκB pathway via the HPK1-C-mediated dominant interfering mechanism (Brenner et al., 2008).

## HPK1-regulated functions are involved in nearly every step of the cancer-immunity cycle

### The Role of HPK1 in Cancer Neoantigen Release

Analysis of the HPK1 literature shows that HPK1 plays a negative regulatory role in nearly every step of the cancer-immunity cycle. The first step of the cancer immunity cycle involves the release of cancer neoantigens (Chen and Mellman, 2013). The release of neoantigens could occur from the spontaneous death of cancer cells through apoptosis, hypoxia-induced necrosis or by chemo/radiation therapy. Some immune-edited cancers that downregulate their MHC Class I molecule as part of their immune evasion tactic may be subjected to lysis by natural killer (NK) cells that become activated through the absence of inhibitory signals normally provided the MHC Class I molecules or by the presence of stress markers MIC-A or MIC-B on cancer cells (Long et al., 2013; Campbell and Hasegawa, 2013). In this regard, NK cells isolated from catalytically inactive K46M HPK1 mutant mice were shown to possess increased cytotoxic activity in vitro against YAC-1, a NK-sensitive murine lymphoma, when compared to the activity of wild type NK cells (Liu et al., 2019). If such increased cytotoxic activity levels were shown to occur in the K46M or K46E HPK1 tumor-bearing hosts, the elevated NK-mediated tumor lysis might increase the amount of neoantigen present for dendritic cells to cross-present to T cells in vivo.

In the case of virus-mediated transformed cancer cells, viral proteins present on the budding virus could be recognized by antibodies. The FcγRIIIa receptor (CD16a) that are present on NK cells could then recognize the antibody-decorated cancer cells and enable NK cells to execute the antibody-dependent cell-mediated cytotoxicity (ADCC). While there is no direct evidence to suggest that HPK1 is involved in ADCC, there is evidence that the loss of HPK1 kinase activity correlates with higher titers of IgG1 and IgG2b produced in response to immunization (Liu et al., 2019). This is consistent with the known inhibitory role HPK1 plays on the induction of phosphorylation-dependent ubiquitination and degradation of BLNK, a B cell-specific homologue of SLP-76 adapter protein that serves one of the critical signalosomes downstream of the BCR-generated signal transduction pathway (Wang et al., 2012b). Therefore, it is reasonable to speculate that an elevated antibody immune response to viral antigen present on virus-transformed cancer cells might increase the potency of ADCC carried out by K46M or K46E HPK1 mutant NK cells. While there is a need to validate the inhibitory role of HPK1 in the NK-mediated release of neoantigens, the observation that the K46M NK cells possess increased cytotoxic activity against YAC-1 cells in vitro suggests that this is a HPK1 kinase-dependent process and thus the enhanced cytotoxic activity of NK cells might be amendable to modulation by small molecule kinase inhibitor of HPK1.

### The Role of HPK1 in Cancer Neoantigen Presentation

The first evidence that HPK1 is involved in the second step of cancer-immunity cycle came when it was shown that *HPK1^-/-^* bone marrow derived dendritic cells (BMDCs) exhibited elevated levels of CD80 and CD86 in response to receiving maturation signal via LPS stimulation (Alzabin et al., 2009). These *HPK1^-/-^* BMDCs also demonstrated increased levels of IL-12 production, reduced levels of IL-10 and an enhanced ability to present peptide antigen. Ovalbumin immunized *HPK1^-/-^* BMDCs could elicit a greater proliferative response of adoptively transferred OTI T cells than their wild type counterpart. This finding is consistent with the report of an elevated OTI proliferative response to enhanced antigen presentation by ovalbumin peptide-pulsed catalytically-inactive K46M HPK1 BMDCs in vitro, which suggests the enhanced antigen presentation is dependent of the HPK1 kinase activity (Liu et al., 2019). In addition to possessing a superior ability to prime T cells, another mechanism might be enhanced antigen presentation in the tumor microenvironment (TME). Given that the *HPK1^-/-^* BMDCs were resistant to LPS-mediated activation-induced apoptosis and *HPK1^-/-^* T cells are also resistant to PGE_2_-induced apoptosis (Alzabin et al., 2009; Alzabin et al., 2010), it is possible that the potent anti-cancer immunity induced by tumor-pulsed *HPK1^-/-^* BMDCs is due in part to their ability to resist apoptosis induction by death receptor ligands or apoptosis-inducing factors present in the tumor microenvironment (TME) (Alzabin et al., 2009). While these data suggest that HPK1 negatively regulates cancer antigen presentation by BMDCs, attribution of its effect on in vivo cancer antigen presentation function to the HPK1 kinase activity could not be made in the studies using *HPK1^-/-^* mice since the absence of the HPK1 protein precludes the possibility of BMDCs generating the caspase-cleaved C-terminal fragment known to facilitate apoptosis by blocking the induction of NFκB activation (Brenner et al., 2009). In fact, Hernandez et al. had argued that the ability of the adoptive transferred catalytically-inactive K46E HPK1 mutant T cells to acquire an enhanced activated phenotype in vivo through priming by the host’s wild type dendritic cells suggests that disruption of HPK1-regulated kinase activity in T cells is sufficient to confer the enhanced tumor immunity phenotype independent of the contribution from enhanced antigen presentation by the kinase inactive K46E HPK1 dendritic cells (Hernandez et al., 2018). However, since dendritic cells from neither the K46E HPK1 mice used by Hernandez et al. nor the dendritic cells from the K46M HPK1 mice used by Liu et al. were assessed for their ability to function in a cancer vaccine the way analogous *HPK1^-/-^* BMDCs were assessed (Liu et al., 2019; Hernandez et al., 2018), the relevance of HPK1 kinase activity in the regulation of cancer neoantigen presentation in the TME warrants further investigation.

### The Role of HPK1 in T Cell Priming and Activation

T cell activation occurs during the third stage of the cancer-immunity cycle. The initial clue that the kinase activity of HPK1 plays a role in inhibiting TCR-induced activation signal came when it was reported that the ectopic expression of HPK1 in Jurkat T cells reduced TCR-induced Erk MAPK and AP-1 activation while the expression of a catalytically inactivated point mutant form of HPK1 could not (Liou et al., 2000). This early finding is consistent with our early observation that Jurkat cells, when treated with Sunitinib malate (Sutent), could produce more IL-2 in response to TCR engagement, resulting in enhanced phosphorylation of Erk MAPK and elevated IL-2 production [unpublished observation and (Johnson et al., 2019)]. Sutent, the multi-kinase inhibitor was capable of inhibiting HPK1 kinase activity in vitro at an IC_50_ of 15 nM (Anastassiadis et al., 2011; Davis et al., 2011). However, the promiscuity of Sutent prevented one from drawing a firm conclusion that the enhanced IL-2 response to TCR activation was exclusively due to the inhibition of HPK1 kinase activity.

Genetic ablation of the HPK1 locus provided additional evidence supporting the role of HPK1 in the negative regulation of TCR-induced T cell activation. The loss of HPK1 correlates strongly with the heightened level of T cell activation as evidenced by increased proliferation, sustained TCR-induced calcium flux and elevated levels of pro-inflammatory cytokines such as IL-2, TNF-α and IFN-γ (Alzabin et al., 2010; Shui et al., 2007). *HPK1^-/-^* mice also possess an enhanced immunopathologic response to myelin oligodendrocyte glycoprotein (MOG) in a murine experimental autoimmune encephalomyelitis (EAE) model (Shui et al., 2007). The severe pathology scores exhibited by *HPK1^-/-^* mice suggested that the loss of HPK1 might have relaxed one or more of the T cell tolerance mechanisms, leading to more severe autoimmune phenotype. These murine studies were consistent with a report that HPK1 expression was reduced in CD4^+^ T cells of systemic lupus erythematosus patients due to jumonji domain containing 3 (JMJD3)-mediated H3 lysine 27 trimethylation (H3K27me3) of the HPK1 promoter (Zhang et al., 2011). A similar report of the reduction of HPK1 expression was detected by microarray analysis of RNA prepared from PBMC of psoriatic arthritis patients (Batliwalla et al., 2005; Stoeckman et al., 2006). While HPK1 expression level is reduced in lupus patients and psoriatic arthritis patients, no conclusion could be drawn from these data as to whether these are biological effects caused by the loss of the scaffolding function provided by the HPK1 protein or were caused by the absence of the HPK1 kinase activity. Further research is needed to establish the role of HPK1 kinase activity in the maintenance of tolerance and prevention of autoimmune diseases (Chuang et al., 2016; Sawasdikosol et al., 2012).

The first evidence that the loss of HPK1 has a positive impact on T cell response to cancer in vivo came when Alzabin et al. demonstrated in the Lewis lung carcinoma model that adoptive transfer of *HPK1^-/-^* T cells is sufficient to confer host mice with the ability to reject the 3LL lung carcinoma, revealing the importance of HPK1 in the control of the T cell intrinsic response to cancer antigens (Alzabin et al., 2010). The definitive proof that the kinase activity of HPK1 negatively regulates T cell activation came when two independently-derived lines of genetically modified mice – the catalytically-inactive K46M and K46E HPK1 point mutants – were assessed for their ability to respond to activation signals: by antibody-mediated TCR-crosslinking, by immunization with model antigens and as well as by in vivo tumor challenge (Liu et al., 2019; Hernandez et al., 2018). T cells from both the K46E and K46M mutant mice failed to phosphorylate SLP-76 at serine 376 and responded to TCR engagement with elevated activation of the Erk MAPK pathway and coincided with higher levels of IL-2 and IFN-γ production when compared to wild type T cells. When the K46E mice were immunized with an ovalbumin-anti-DEC205 conjugate as a model antigen, targeting ovalbumin to DEC205^+^ dendritic cells for cross-presentation priming of CD8^+^ T cells, it was observed that a higher percentage of OVA peptide primed SIINFEKL^+^ CD8 T cells expressed elevated levels of IFN-γ- and granzyme B, despite the lack of a significant increase in SIINFEKL^+^ CD8 T cells (Hernandez et al., 2018). These data suggest that there is no proliferative advantage when the HPK1 kinase activity is absent, however, T cells demonstrate enhanced activation. In contrast, when catalytically inactive K46M HPK1 mutant mice were immunized with ovalbumin, a higher percentage of BrdU-incorporated CD45^+^, CD4^+^, CD3^+^ splenic and lymph node T cells were found in the immunized K46M HPK1 mice than in the wild type cohort (Liu et al., 2019). These data suggest that the absence of HPK1 kinase activity correlates with increase T cell proliferation, as indicated by increase in BrdU uptake. The enhance proliferative response observed in the K46M HPK1 mice could be due to the enhance proliferative response of CD4^+^ K46M HPK1 T cells to ovalbumin, which is not captured in the response of K46E HPK1 mice to ovalbumin-anti-DEC205 vaccination. Additional studies are needed to resolve this apparent paradox.

### The Role of HPK1 in T Cell Trafficking to Tumor

Neoantigen-primed activated T cell clones need chemotactic signals to guide its migration towards the tumor. There is no direct evidence that HPK1 plays a significant role in this fourth stage of cancer-immunity cycle. With regard to the possible involvement of HPK1 in the regulation of T cell trafficking to the tumor, CX3CL1 transcripts are found to be elevated in tumor draining lymph nodes of tumor-bearing K46M *HPK1* mice (Liu et al., 2019). High levels of CX3CL1 is known to be associated with an increased number of CD8^+^ and CD4^+^tumor infiltrating lymphocytes (TILs) in colorectal cancer and is a good prognostic indicator of survival (Franciszkiewicz et al., 2012). While there is no direct evidence to implicate HPK1 in CX3CL1-mediate chemotaxis, reports of increased CD4^+^ and CD8^+^ TILs in tumors grown in K46M mice and increased CD8+ TILs in tumors grown in K46E mice allow us to infer that the loss of HPK1 kinase activity might be contributing to the increased chemokine-mediated chemotaxis towards tumors (Liu et al., 2019; Hernandez et al., 2018).

### The Role of HPK1 in Tumor Infiltration

It is during the fifth stage of cancer-immunity cycle that the antigen-primed CD8^+^ cytotoxic T lymphocytes have to exit the vasculature and extravasate towards the tumor, as they are continually summoned to migrate towards the increasing concentration of the chemokine gradient. This multi-step process requires the binding of the β2 integrin, the Lymphocyte Function-Associated antigen 1 (LFA-1), to its cognate ligand, the intercellular cell adhesion molecule-1 (ICAM-1) that is expressed on the endothelial cells (Slaney et al., 2014). The LFA-1 affinity for ICAM-1 is regulated by the signals provided by the TCR and chemokine receptors via a complex formation between the LFA-1 and the adhesion and degranulation promoting adapter protein (ADAP), forming an indispensable supramolecular complex of SLP-76, SKAP55 and Rap1 GTPase that translocate to the plasma membrane to activate LFA-1 (Slaney et al., 2014; Baker et al., 2009; Wang et al., 2014). HPK1 negatively regulates the TCR-initiated LFA-1 activation by binding to the SH2 domain of SLP-76, limiting the available pool of SLP-76 to form the supramolecular complex with ADAP (Wang et al., 2004). In this model, there is no evidence that HPK1 mediates its negative regulatory function via its kinase activity, but rather HPK1 uses its protein scaffolding ability to compete with and to disrupt the formation of the ADAP-based LFA-1 activation complex (Patzak et al., 2010). Similar HPK1-based mechanism might also be operating in B cell adhesion where HPK1 is complexed with a SKAP-HOM, a SKAP55 homologue (Königsberger et al., 2010). Translational silencing of HPK1 by shRNA resulted in an increased Rap1 activation and cell adhesion to ICAM-1 (Königsberger et al., 2010). While these studies provided evidence that HPK1 functions as negative regulator of LFA-1-dependent cellular adhesion in T and B cells, HPK1 assumes a diametrically opposed role of being an essential activator of the CXCL1-induced LFA-1 activation of polymorphonuclear neutrophils, enabling neutrophils to adhere to ICAM-1-expressing cells and extravasate into tumors (Jakob et al., 2013). While HPK1 appears to utilize a non-kinase function to regulate LFA-1 through competition with ADAP, one cannot rule out the possible involvement of HPK1 kinase activity in this process since none of the studies were conducted using catalytically-inactivated K46M and K46E HPK1 mutant T cells.

### The Role of HPK1 in Cancer Cell Recognition and Engagement

In the sixth and seventh stages of the cancer-immunity cycle, TILs are challenged by an inhospitable TME. Apoptosis-inducing receptors such as Fas-L or the ligand for the checkpoint inhibitors such as PD-L1 are two examples of numerous receptors that tumor cells co-opt to inhibit the CD8^+^ TILs (Vinay et al., 2015; Kim et al., 2006). In addition to the receptor-based apoptosis-inducing challenges that TILs have to overcome, tumors also make soluble immune suppressive factors ranging from cytokines such as TGFβ, to inhibitory lipid mediators, such as PGE_2_, and nucleosides, such as adenosine, to induce T cell inhibitory signals (Greenhough et al., 2009; Allard et al., 2016). In addition to factors that are directly produced by the tumor, suppressive immune cells, such as regulatory T cells (Tregs) and myeloid-derived suppressor cells (MDSCs), are also attracted to the inflammatory signals generated by the tumor (Wing et al., 2019; Motallebnezhad et al., 2016). Suppressor cells contribute significantly to negatively effect the TILs’ ability to control tumor growth. In addition to the plethora of immunosuppressive mechanisms to suppress TILs’ cytolytic activity, TILs may also encounter immunoedited tumors that have evolved to escape T cell surveillance by down-regulating its MHC Class I molecules (Garrido and Algarra, 2001). These cancer cells may still produce neoantigens that could be picked up by antigen presenting cells and be cross-presented to CD8^+^ T cells, but the primed and activated CD8^+^ TILs are unable to engage these cancer cells due to the absence of the MHC Class I. In this situation, the immune system has to rely on cell types other than T cells to engage cancer cells that do not express MHC Class I molecules. MHC Class I also functions as inhibitory receptors for NK cells, thus the loss of MHC Class I molecules could allow NK cells to be activated, enabling them to engage MHC Class I-deficient cancer cells (Long et al., 2013; Campbell and Hasegawa, 2013).

Studies using genetically modified mice suggests that T cells that do not express HPK1 or express the catalytical inactive form of HPK1 are resistant to many of these immunosuppressive factors. These HPK1 mutant T cells have significant resistance to PGE_2_-mediated and adenosine-mediated inhibition of TCR-induced proliferative responses, as well as to the suppression of TCR-induced IL-2 production (Liu et al., 2019). K46M HPK1 mutant mice could effectively control the growth of a syngeneic sarcoma tumor (1956) that produces high levels of both PGE_2_ and adenosine. Nanostring analysis of tumor draining lymph nodes from K46M mice that harbor the 1956 tumor revealed that pathways known to associate with the apoptotic network are down-regulated (Liu et al., 2019). This collection of findings is consistent with the previous report that *HPK1^-/-^* T cells are resistant to PGE_2_-mediated suppression of TCR-induced proliferative responses and IL-2 production, as well as being resistant to PGE_2_-mediated apoptosis (Alzabin et al., 2010). As with the K46M mice, *HPK1^-/-^* mice could effectively control the of PGE_2_-dependent growth of syngeneic lung carcinoma tumor (3LL), suggesting that the resistance to PGE_2_-mediated inhibition may contribute to effective tumor control (Alzabin et al., 2010).

The catalytically inactive K46E and K46M HPK1 mutant mice successfully control the growth of GL261 glioma and sarcoma 1956, respectively (Hernandez et al., 2018; Liu et al., 2019). K46E mice responded to GL261 tumor with increased CD8^+^ T cell infiltrates that expresses elevated levels of IFN-γ and TNF-α. This elevated anti-tumor immunity correlated with a 90% response rate in the control of tumor growth. Similarly, the K46M HPK1 mutant mice were able to effectively slow the rate of 1956 tumor growth. Analysis of TILs in this model revealed a significant reduction of Treg infiltrates and thus a corresponding increase in the CD8^+^ to Treg cell ratios. Quantitative PCR and nanostring analysis of RNA isolated from these tumors revealed increased immune cell markers involved in mediating anti-tumor immunity, most notably CD4, CD8, IFN-γ, TNF-α, granzyme B, CD28, CD11c and CD11b are elevated in K46M mice. Taken together, the results of these studies presented a convincing set of pre-clinical data confirming the importance of HPK1 kinase activity in the inhibition of effective anti-tumor immune response.

T cells that have undergone chronic immune engagement against viral infection or cancer become less capable of engaging these targets as well as they once did when they first encounter them. These T cells undergo phenotypic and functional changes that are recognizably distinct from that of functional effector or memory T cells (Wherry, 2011; Blank et al., 2019). This ‘exhausted’ phenotype is characterized functionally by poor effector function and sustained expression of inhibitory receptors, including PD-1. To assess whether HPK1 kinase activity plays a role in regulating T cell exhaustion, the K46E HPK1 mutant mice were infected with the Clone 13 strain of lymphocytic choriomeningitis virus (LCMV) and found to responded with reduced viral titers in blood, kidney and liver, which correspond with nearly a two fold increase compared to wild type mice in the frequency of LCMV-specific GP276^+^ CD8 T cells (Hernandez et al., 2018). The superior control of LCMV infection by the K46E HPK1 mutant mice without a corresponding decrease in PD-1 levels suggests that the exhaustion phenotype as defined by PD-1 expression level is not diminished in K46E mice, suggesting that the co-administration of anti-PD-L1 therapeutic antibody might further improve the enhanced viral immunity. With the PD-1/PD-L1 axis blocked by the anti-PD-L1 antibody, the K46E mice cleared the LCMV virus to levels below the sensitivity of detection, confirming that the K46E T cells were still sensitive to PD-1/PD-L1-mediated inhibition (Hernandez et al., 2018). The enhanced response of the K46E mice to anti-PD-L1 therapy is likely to be due to superior priming and activation that occurs during the early phase of viral infection, prior to the initiation of the exhaustion phenotype brought about by chronic infection. Thus, the inhibitory pathways regulated by HPK1 is distinct from that of PD-1, which suggests that a small molecule inhibitor of HPK1 could be used in conjunction with PD-1/PD-L1 inhibition.

The presence of Treg is a prognostic indicator of poor disease outcome in mouse tumor models and in cancer patients because of their potent ability to inhibit T cell functions (Nishikawa and Sakaguchi, 2010; Facciabene et al., 2012). Whether HPK1 plays a role in the inhibitory functions of Treg could have an immense impact on tumor immune responses. For this reason, the homeostatic level of *HPK1^-/-^* Tregs were assessed and found to be elevated when compared to the wild type Tregs (Sawasdikosol et al., 2020). This surprising finding could be the result of a positive feedback mechanism trying to elevate the Tregs numbers in order to compensate for the possible loss of their suppressive function. Further analysis revealed that *HPK1^-/-^* Tregs have reduced ability to inhibit TCR-induced proliferative responses by effector T cells. In addition, *HPK1^-/-^* Tregs respond to TCR engagement with an elevated and sustained Erk MAPK and p65/RelA NF-κB phosphorylation, which correlate with their ability to produce IFN-γ, CCL3, CCL4 and IL-2. This atypical phenotype occurs in the presence of normal FoxP3 expression levels and is most consistent with the phenotype of ‘fragile’ Treg (Overacre-Delgoffe and Vignali, 2018; Overacre-Delgoffe et al., 2017). While the functional properties of K46M HPK1 mutant Tregs have not been characterized, the K46M HPK1 mutant mice bearing the 1956 tumors possess a lower percentage of Treg relative to CD45^+^ TILs, causing an increase in the CD8^+^ effector T cells:Tregs ratio (Liu et al., 2019). This reduced Treg numbers among K46M TILs might contribute towards the enhanced control of 1956 growth exhibited by these mice. Whether the K46M mice maintain the suppressive ability comparable to those of wild type Tregs remains to be determined.

## Complexities associated with targeting HPK1 with small molecule kinase inhibitors

HPK1 may have the potential to become a new IO drug target as suggested by the genetic evidence. These data have stimulated a large number of pharmaceutical companies, as well as academic research institutions, to undertake pre-clinical research focusing on the development of small molecule kinase inhibitors of HPK1. While a large number of HPK1 programs have identified lead HPK1 kinase inhibitors that can block HPK1 kinase activity with the half maximal inhibitory concentration IC_50_ values in the low nM concentration, there have been no published report of enhanced anti-tumor immunity that have been elicit by small molecule inhibitor of HPK1 to date. Given that there are quite a number of Ste20 family members that function as MAP4Ks and share high sequence homology of their kinase domains with HPK1, it is extremely challenging to develop a highly selective small molecule that does not inhibit one or more of the closely related Ste20 family member of HPK1. Some of these members have been shown to play a positive role in anti-tumor immunity (Chuang et al., 2016).

The recently solved structures of HPK1 kinase domain, along with the existing structure of other HPK1 family members, should facilitate the iterative improvement through structure-activity relation-driven modifications (Wu et al., 2019; Johnson et al., 2019). The uncommon domain-swapped dimeric conformation and the structural changes accompanying HPK1 trans-regulation revealed by these studies may facilitate future insights regarding the strategy to enhance selectivity of the small molecule inhibitor of HPK1. (Wu et al., 2019; Johnson et al., 2019; Sawasdikosol and Burakoff, 2019). As an alternative approach, large pharmaceutical companies with sufficient resources might engage in an unbiased high-throughput screen of their small molecule libraries for allosteric inhibitors of HPK1. Such inhibitors might possess a lower propensity to unintentionally hit other kinases since allosteric inhibitors are likely to be inhibiting HPK1 kinase activity by binding to a non-ATP-competitive binding site that might be uniquely present on HPK1.

It is clear that developing selective kinase inhibitors as an IO drug poses a significant challenge for the drug discovery teams than would the typical screen for small molecule drugs designed to block kinase drivers of cancer growth. In the latter scenario, if the oncogenic kinase being targeted is a *bona fide* primary driver of tumor growth, blocking the catalytic activity with small molecule inhibitors should result in the elimination of tumor, or should at least retard its growth, regardless of what other kinases the inhibitors might be hitting unintentionally. In this scenario, the off-target liability screen of the drug is focused on minimizing the adverse health impacts on the life-sustaining functions. However, in the case of HPK1 inhibitor, the bar for acceptable off-target liability is significantly higher since the mechanism of action of the HPK1-based IO drug depends on provoking the dormant or exhausted immune system to engage cancer. This indirect mechanism of cancer engagement through immune system modulation necessitates that compounds targeting HPK1 inhibitor clear the same safety standards imposed on the traditional oncology drug, and it must also avoid off-target impacts that could interfere with the normal functions of the immune cells that are supposed to be executing the killing of the cancer cells. Having to screen compounds against off-target liabilities that could negatively impact the immune response adds an additional layer of complexity to the drug developmental process (Sawasdikosol and Burakoff, 2019).

Exclusive reliance on the inhibition of HPK1 kinase activity to achieve an anti-tumor immune response leaves in place an HPK1 molecule that may have other functions that inhibit the immune response. Given the involvement of the scaffolding properties of HPK1 in a number of critical immunological processes such as NFκB regulation during AICD and LFA-1 activation, it is possible that complete elimination of the HPK1 protein is required to achieve optimal anti-tumor immune response. The fact that *HPK1^-/-^* mice appeared to reject 3LL tumors far more effectively than the catalytically inactive HPK1 mutant mice could reject other syngeneic tumors supports the possibility that a complete removal of HPK1 protein is required for the maximal anti-tumor immunity outcome. Should further studies show that the complete removal of HPK1 is a superior approach to confer immune cells with more robust anti-tumor immunity, a number of approaches are available to eliminate the protein, such as clustered regularly interspaced short palindromic repeats (CRISPR), short hairpin RNAs (shRNA), antisense oligonucleotides (ASO) or Proteolysis Targeting Chimeras (PROTACs).

Lastly, there remains one other possible obstacle for targeting HPK1. While it is generally believed that HPK1 is expressed at high levels only in hematopoietic cells, its expression is not exclusively restricted to hematopoietic cell compartments. Given that HPK1 transcripts are found is all embryonic tissues, it may not be surprising that transcripts have been found in non-hematopoietic cell lineages, including brain and testes (Kiefer et al., 1996). Recently, HPK1 has also been shown to be expressed and associated with the oncogenic receptor tyrosine kinase, AXL in early pancreatic intraepithelial neoplasia (PanIN) (Wang et al., 2009; Song et al., 2020). As PanIN progresses to a more aggressive pancreatic ductal adenocarcinomas (PDAC), HPK1 expression is loss via CUL7/Fbxw8 ubiquitin ligase-dependent targeting of HPK1 for degradation via the 26 s proteasome (Wang et al., 2009; Wang et al., 2014). The presence of HPK1 promotes the increased association between AXL and the c-Cbl ubiquitin ligase, and the increased association between these two molecules correlates with AXL ubiquitination and downregulation. Reconstituting HPK1 expression in PDAC cells causes PDAC cells to undergo cell cycle arrest and growth inhibition. These results suggest that HPK1 may be functioning as a tumor suppressor gene in PanIN cells. If so, therapeutic approaches that either inhibit HPK1 kinase activity or reduce HPK1 protein levels in PanIN cells may produce an unintended consequence of promoting the transitioning of PanIN to a more aggressive PDAC cells. Because HPK1 could be expressed in non-hematopoietic cells and might function as a tumor suppressor gene, cancer cells should be screened for the expression of HPK1 and use HPK1 expression as a biomarker to determine whether the use of a HPK1 targeted IO drug would be appropriate for that particular tumor sub-type.

## Conclusion

The prospect of targeting HPK1 with a small molecule, raises the possibility of developing a class of small cytosolic IO drug with potential oral availability. While the interest in HPK1 rests on solid genetic evidence obtained from murine tumor models, a note of caution is warranted in that, to date, there is no known human disease linked directly to a mutation or deletion of the *HPK1* locus. Correlations between reduced HPK1 expression levels and autoimmune diseases such as systemic lupus erythematosus (Zhang et al., 2011) and psoriatic arthritis (Zhou et al., 1999) have been reported, but there is no direct evidence to suggest that these individuals have enhanced anti-tumor immune responses due to decreased HPK1 protein levels. Also, it is possible that mutated *HPK1* alleles conferring superior immune surveillance and control of nascent cancer exists, but we have failed to take notice of these individuals since such functional changes in tumor immunity is not necessarily deleterious to one’s health, unless it is associated with severe, life-threatening autoimmune diseases. Given the absence of human genetic evidence for the role of HPK1 as a negative regulator of tumor immunity and the existing precedence that not all therapeutics developed in murine tumor models deliver similar success in cancer patients, the fate of HPK1-based IO therapy ultimately rests on the outcome of human clinical trials.
